# Wounds of the Eye

**Published:** 1910-12

**Authors:** A. W. Stirling

**Affiliations:** Ophthalmic Surgeon to the Grady Hospital


### WOUNDS OF THE EYE.

By A. W. Stirling, M. D., M. B., C. M. (Edin.), D. P. H. (Lond,)

*Ophthalmic Surgeon to the Grady Hospital.*

The following paper I have written with the idea that it might probably be useful to medical men who make no profession of being experts in ocular diseases. Most wounds of the eye pass in the first instance into the hands of general practitioners, with whom, therefore, the ultimate outcome in a great measure lies. I began to cite cases illustrative of each of the conditions discussed, but soon found that the dissertation would thereby be inordinately prolonged, and therefore, fell back on the more generalized method of discussion.

Foreign bodies, when minute, are occasionally difficult to see, but oblique illumination, and if necessary, the use of fluorescine, will usually suffice to discover their presence. There is, however, often none when the patient is ready to take oath that there is. In their removal antiseptic precautions should be employed and mydriasis and a bandage for twenty-four hours are often advisable, because serious infection is not uncommon following upon a simple abrasion of the cornea. When deeply placed in the cornea, it may be necessary to pass a broad needle into the anterior chamber in order to prevent the foreign body from being pushed through Descemet's membrane.

When strong acid has entered the eye, bicarbonate of soda may be used to neutralize it, and boric acid will neutralize alkalies, while castor oil locally is comforting; but whatever may be escharotic, it is well to remember that it is easier to prevent adhesions than to cure them. A glass rod and lots of vaseline may thus prove useful. There are metal burns and lime burns, which may appear at first equally hopeless. It is usually safe, however to give a better rognosis in the metal than in the lime cases, as the former are apt to do better and the latter worse than is expected. Of course, all foreign substance must be carefully removed after the concainisation.

Contusions are responsible for all degress of trouble from the too suggestive but ephemeral—black eyes, to total loss of vision. After even the least serious-looking wound it is wise to be chary of an off hand dismissal of the case as of no serious moment, for some of the results of blows to the eye are not always apparent at first. Simple corneal abrasions should be cleaned and watched for a few days, a bandage having been applied.

I have seen deep opacities due to a wrinkling of the posterior membrane of the cornea, and in the corneal substance, probably due to aggregations of lymph between the lamellae. These disappear with time, but actual ruptures of the posterior membrane, which then curls up, of course, do not. When the globe is ruptured, it is generally by an oblique wound passing from without, inwards and forwards.

The iris will then probably be separated partially or it may be lost outside the eye or be seen in the bottom of the anterior or the vitreous chamber.

The lens may be dislocated in similar directions, while it may lie outside the wound, but beneath a still intact conjunctiva.

Very often these wounds necessitate excision of the eye, but sometimes the edges may be brought together by sutures. It is usually rcommended that prolapsed iris should be excised, but cases sometimes appear to be better left alone eserine an atrophine being used with the object of removing the prolapse, but where the lens lies beneath the conjunctiva the lens should be untoucher, at least til the deep wound has healed.

The iris may be wounded without rupture of the globe. The pupil may then be dilated and stationary, owing to paralysis of the third nerve, though tears at either periphery of the iris are commonly present. The iris may alse be wholly or more often partially inverted toward the ciliary body, the part inverted disappearing.

In all these wounds cold compresses are useful, and atrophine should be employed, but not in ruptures of the internal edge of the iris.

The ciliary body may be so affected that paralysis of accommodation results, and the refraction of the eye may be changed. The latter may, of course, be due to slight dislocation of the lens or to altered curvature of the cornea, but I have seen a hypermetropic turned to a myopic eye without any other visible alteration. Subluxation of the lens produces much astigmatism, possibly monoculerdiplopia, and when the lens lies in the anterior chamber irido-cyclitis and also glaucoma are common results. The lens should then if possible be removed, and when it lies in the anterior chamber this should in every case be done. I have seen a lens which its owner could pass at will from one chamber to the other, lie there for long, apparently harmless, only to set up glaucoma in the end. It must be borne in mind that the lens capsules may be ruptured as usual, near the periphery, without present symptoms. But the lens before long become cataractous. The vitreous after a blow may be found opaque:—a hemorrhage has taken place; or it may be gradually filled by floating opacitis as a result of disease of the uveal tract. When blood in the vitreous has been absorbed one or more white arcs may sometimes be seen, concentric with the optic disc and with vessels passing over them. These are ruptures of the choroid, showing the sclera.

Rupture of the retina is very rare, but its detachment is quite common. Commotio Retinae shows as a milkiness near the optic disc, probably an oedema, and likely to disappear within a few days, leaving the vision as before. Much more serious and not unlikely to be overlooked by the careless observer, is the so-called "hole" at the macula, while instead of the punched-out appearance an aggregation of dark spots may appear in the same locality, associated with uncreasingly defective vision.

Wounds from sharp instruments may, of course, be of any depth and in any position. When in the conjunctiva only, they soon heal with or without stitches. Corneal wounds may heal quickly, leaving behind, however, a scar, and sometimes astigmatism. But if they are large, and especially, if the sclera is also cut, and where the lens is involved, serious complications are probable. Prolapse of the iris, if seen within an hour or two, may be reduced by atrophine or eserine, otherwise it is usually adsuch action depends on the possibility of reducing it. If the prolapse is cauterized danger is minimized. Adhesions of the iris to the wound promotes an irritation which is detrminental to healing.

Such adhesion whenever possible should be divided by Lang's Twin Knives. The swelling of a wounded lens irritates the isis, increases the danger of infection, and adhesion of the iris to the wound promotes an irritation which is detrimental to healing. and interferes with the formation of the anterior chamber, which also becomes filled by soft lens matter. A favorable vidus is thus afforded, in which germs may multiply. Either panopthalmitis of sympathic opthalmia is specially to be feared in such cases. Secondary glaucoma must also be expected, and one should be prepared at any to to evacuate the contents of the anterior chamber.

When the wound is large, and much vitreous as well as iris and perhaps ciliary bodies are prolapsed, it is quite improbable that any useful amount of vision will be retained, and the eye had better be excised, in order to avoid the risk of sympathetic opthalmia. If after a careful examination of the eye and concerning the instrument as regards sterility, it seems that the attempt is worth while, the eye having been well cleansed, fine sutures should be used in the sclera and the conjunctiva afterwards closed over the wound, all prolapsed tissue having been carefully removed. Atropine is instilled and complete rest in bed enjoined. If, at the end of a week, the eye is not quieting, it had better be excised, and for a considerable period both corneae must be closely watched for a deposit of so-called keratitis punctata.

The effects of puncture of the lens capsule vary according to the size of the wound. When quite small the capsule will probably heal and the opacity caused by the imhibition of aqueous or vitreous fluid may remain limited.

Usually the opacity spreads, and the capsule must be reopened in young people, in order that the lense substance may gradually escape and be absorbed, as will happen when the original wound is large. But if the lens swells and escapes too rapidly it will block the lymph circulation through the anterior chamber, irritate the iris and ciliary body and necessitate the opening of the anterior chamber in order to avoid iritis and cyclitis as well as secondary glaucoma, with resulting atrophy of the optic nerve. The pupil must be well dilated in order to keep the iris out of the way of pressure and free from the prospect of adhesion to the lens.

When an ocular wound has resulted from a flying foreign body of small size, minute examination with a magnifying glass for perforation of cornea, sclera and iris must have been made before one is justified in asserting that nothing has entered the eye. Foreign bodies, if retained, make the prognosis always bad, and an endeavor should be made to remove them. I have known sympathetic opthalmia to be set up very many years later by a small body in the retina. Iron stains the tissues and is specially apt to irritate. But where no damage has been done to sight, which the operation of removal would destroy, especially when there is reason to believe that the invading particle is sterile, as is often the case with metal heated by the blow of a hammer, it may be preferable not to open the eye.

Ocular wounds often place the surgeon in an exceedingly delicate position, and nowhere are knowledge and judgment more necessary. Time would fail were one to try here to discuss fully the intricacies of this subject. I shall avoid so doing, merely remarking that while the magnet may at the time of operation appear to have given brilliant results the later effects of the traction of metal through the tissues are very apt to be disastrous.

